# Improvements to the gastric cancer tumor-node-metastasis staging system based on computer-aided unsupervised clustering

**DOI:** 10.1186/s12885-018-4623-z

**Published:** 2018-07-03

**Authors:** Zhiqiong Wang, Mo Li, Zhen Xu, Yanlin Jiang, Huizi Gu, Ying Yu, Haitao Zhu, Hao Zhang, Ping Lu, Junchang Xin, Hong Xu, Caigang Liu

**Affiliations:** 1 Sino-Dutch Biomedical and Information Engineering School, Northeastern University, Shenyang, 110169 China; 2 Department of General, Visceral and Transplantation Surgery, Section Surgical Research, University Clinic Heidelberg, Im Neuenheimer Feld 365, 69120 Heidelberg, Germany; 3 Department of Breast and Thyroid Surgery, Affiliated Zhongshan Hospital of Dalian University, Dalian, 116001 China; 4 Department of Internal Neurology, the Second Hospital of Dalian Medical University, Dalian, 116027 China; 5 Liaoning Medical Device Test Institute, Shenyang, 110179 China; 6 Department of Gastric Surgery, Liaoning Cancer Hospital and Institute, Cancer Hospital of China Medical University, Shenyang, 110042 China; 7 Department of Breast Surgery, Liaoning Cancer Hospital and Institute, Cancer Hospital of China Medical University, No. 44, Xiaoheyan Road, Dadong District, Shenyang, 110042 Liaoning Province China; 8 Department of Surgical Oncology, the first hospital of China Medical University, Shenyang, 110001 China; 9 School of Computer Science and Engineering, Northeastern University, Shenyang, 110189 China; 10 Department of Breast Surgery, Shengjing Hospital of China Medical University, Shenyang, 110004 China

**Keywords:** Gastric cancer, Tumor-node-metastasis staging, Computer-aided unsupervised clustering method

## Abstract

**Background:**

The Union for International Cancer Control (UICC) tumor-node-metastasis (TNM) classification is a key gastric cancer prognosis system. This study aimed to create a new TNM system to provide a reference for the clinical diagnosis and treatment of gastric cancer.

**Methods:**

A review of gastric cancer patients’ records was conducted in The First Hospital of China Medical University and the Liaoning Cancer Hospital and Institute. Based on patients’ prognoses data, computer-aided unsupervised clustering was performed for all possible TNM staging situations to create a new staging division system.

**Results:**

The primary outcome measure was 5-year survival, analyzed according to TNM classifications. Computer-aided unsupervised clustering for all TNM staging situations was used to create TNM division criteria that were more consistent with clinical situations. Furthermore, unsupervised clustering for the number of lymph node metastasis in the N stage led to the formulation of a classification method that differs from the existing N stage criteria, and unsupervised clustering for tumor size provided an additional reference for prognosis estimates.

**Conclusions:**

Finally, we developed a TNM staging system based on the computer-aided unsupervised clustering method; this system was more in line with clinical prognosis data when compared with the 7th edition of UICC gastric cancer TNM classification.

## Background

In the past 3 decades, both the Japanese and Union for International Cancer Control (UICC) tumor-node-metastasis (TNM) classification systems for gastric cancer have undergone several major changes [1]. The biggest difference between the 2 systems exists in the N stage division method [2]. However, in 2010, the UICC released the 7th edition of TNM classifications of gastric cancer that used the number of metastatic lymph nodes for N classification. This standard has now been adopted by the Japanese TNM [3]. However, the exact threshold values for division between the different N stages have become a critical issue.

In clinical practice, other independent clinical or pathological features can directly or indirectly predict patient survival [4–9]. For example, tumor size, although closely related to the T stage, remains an independent prognosticator in patients with gastric cancer. Therefore, the threshold tumor size and its effect on prognosis need to be evaluated to help clinicians determine patient prognosis more accurately.

Importantly, although TNM staging has been revised several times, in clinical practice, there is often a marked difference in the prognoses of patients with the same TNM stage, which might be owing to heterogeneity between patients of different ethnic backgrounds, the evolution of the biological behavior of gastric cancer, and other factors [10]. Moreover, among patients with a poor prognosis, there are those who achieve long-term survival. Therefore, a more accurate division of the TNM stages is needed to determine patient prognoses, comprehensive treatment planning, and other disease management aspects [11–13].

To resolve the problems mentioned above and develop a system for improved prognostic accuracy, we summarized information obtained from patients with gastric cancer who underwent treatment over the past 3 decades [14]. We conducted a precise enumeration of the optimal division points for clinical factors related to gastric cancer (e.g., age, tumor size, the number of lymph node metastases), and selected the optimal cut-off points. Data permutations were performed to obtain the final TNM staging system based on the principle of having smaller differences within groups and greater differences between groups. The postoperative 5-year overall survival rate was used as the comparison standard to account for the extensive duration of the study period. This study provided a reference for determining more scientific and accurate TNM stage division criteria, as well as threshold values for various factors that might influence gastric cancer prognosis.

## Methods

### Patients

We enrolled 2414 patients with histologically confirmed gastric cancer who underwent surgery at the Liaoning Cancer Hospital and Institute and China Medical University. All patients had complete medical records available.

All patients were followed-up by postal or telephone interviews. The last follow-up was conducted in December 2015, with a total follow-up rate of 91%. Clinical, surgical, and pathological findings, and all follow-up data were collected and recorded in the database.

The study protocol was approved by the Ethics Committee of The First Hospital of China Medical University and the Liaoning Cancer Hospital and Institute, and informed consent was obtained from all subjects. All methods were performed in accordance with the relevant guidelines and regulations.

### Endpoints and follow-up

The primary endpoint was the 5-year survival. Overall survival time was calculated from the date of surgery until the date of death or last follow-up contact. Patient data were censored at the last follow-up when they were alive. Follow-up assessments were conducted every 6 months for the first 5 postoperative years, and every 12 months thereafter until death.

### Computer-aided unsupervised clustering method

A precision enumeration was performed to determine the optimal division points for clinical factors related to gastric cancer (e.g., age, tumor size, the number of lymph node metastasis), and all possible division points were calculated to form a cycle. For each cycle, the log-rank test was used to derive the *p*-value between 2 points. At the end of each cycle, the minimum p-value cut-off point was selected as the optimal cut-off point.

Permutations were carried out for the 5 T stages, 4 N stages, and 2 M stages in TNM gastric cancer staging, i.e., a total of 5 × 4 × 2 = 40 groups. Log-rank test *p*-values between these groups were calculated; differences within groups were minimized, and those between groups were maximized by combining groups with greater p-values into a single unit, thereby, obtaining the 7 most optimal groups as the final TNM stages.

### Statistical analyses

Kaplan-Meier survival curves were used to estimate 5-year overall survival. For univariate analyses, the prognostic factors of interest and the diagnosis period were covariates in the Cox regression model. Multivariate analyses were conducted using the Cox proportional hazards regression model to assess risk factors associated with survival. Two-sided p-values < 0.05 were considered statistically significant. Analyses were performed using SPSS software, version 23.0.

## Results

### Patients

Patient characteristics are shown in Table 1. The median age of patients at gastric cancer onset was 57 years, and there were significantly more male patients compared with female patients. In most patients, the gastric cancer was located in the lower portion of the stomach and presented at an advanced stage. Almost 50% of the patients underwent radical surgery, with the scope of lymph node resection being based on D2 surgery. The results of the multivariate analyses of factors associated with survival are shown in Table 2. After adjusting for 16 variables, patient survival was significantly associated with tumor size, tumor site, gross appearance, T stage, N stage, TNM stage, hepatic metastasis, and peritoneum metastasis. Factors such as the surgical extent and joint organ removal also affected prognoses. Adjuvant chemotherapy and the diagnosis period affected the 5-year overall survival rates.

**Table 1:** Characteristics of population from the three periods (*n* = 2414)

Variable	Subgroups	Frequency (%)
Age at diagnosis (Mean ± SD)		57.49 ± 11.32
Gender	Male	1738 (72.00)
	Female	676 (28.00)
Tumor size(Mean ± SD)		5.66 ± 3.08
Site of tumor	Whole stomach	174 (7.21)
	Upper stomach	263 (10.89)
	Middle stomach	248 (10.27)
	Lower stomach	1243 (51.49)
	> 2/3 stomach	486 (20.13)
Pathological tumour stage (%)	T1	342 (14.17)
	T2	1136 (47.06)
	T3	515 (21.33)
	T4a	208 (8.62)
	T4b	213 (8.82)
Pathological nodal stage (%)	N0	884 (36.62)
	N1	451 (18.68)
	N2	530 (21.96)
	N3	549 (22.74)
TNM stage (%)	IA	272 (11.27)
	IB	394 (16.32)
	IIA	391 (16.20)
	IIB	371 (15.37)
	IIIA	399 (16.53)
	IIIB	237 (9.82)
	IIIC	116 (4.81)
	IV	234 (9.69)
Gross type (%)	Borrmann I	26 (1.17)
	Borrmann II	384 (17.25)
	Borrmann III	1558 (70.02)
	Borrmann IV	257 (11.55)
Surgery (%)	Absolutely curative	1116 (46.23)
	Relatively curative	819 (33.93)
	Palliative	479 (19.84)
Lymph node dissection (%)	D1	238 (9.86)
	D2	1584 (65.62)
	D3	204 (8.45)
	Palliative resection	388 (16.07)
Complication (%)	Intestinal obstruction	56 (2.32)
	Anastomotic leakage	32 (1.33)
	Pneumonia	9 (0.4)
	Abdominal abscess	39 (1.62)
	Anaemia	16 (0.7)
	Other	83 (3.44)
Hepatic metastasis (%)		72 (2.98)
Peritoneum metastasis (%)		178 (7.37)
Adjunctive therapy (%)		475 (19.68)
Type of gastrectomy (%)	Total	403 (16.69)
	Subtotal	2011 (83.31)
Combined organ resection (%)	Pancreas or spleen	159 (6.59)
	Liver or gall	78 (3.23)
	Transverse colon	214 (8.86)
	Other	68 (2.82)
Diagnosis period	1980s	496 (20.5)
	1990s	673 (27.9)
	2000s	1245 (51.6)

**Table 2:** HR for death in population (*n* = 2414) —univariable and multivariable analysis

	Univariable analyses		Multivariable analyses	
	HR (95% CI)	*p* ^a^	HR (95% CI)	*p* ^b^
Age (years)		0.005		0.301
≤ 55	1 (Ref)		1 (Ref)	
> 55	1.180 (1.052–1.322)	0.005	1.066 (0.944–1.204)	0.301
Sex		0.801		0.937
Women	1 (Ref)		1 (Ref)	
Men	1.016 (0.897–1.151)	0.801	0.995 (0.872–1.135)	0.937
Tumor size		0.000		0.000
≤ 4 cm	1 (Ref)		1 (Ref)	
5–8 cm	2.101 (1.848–2.389)	0.000	1.256 (1.091–1.446)	0.001
≥ 9 cm	3.694 (3.152–4.328)	0.000	1.372 (1.117–1.686)	0.003
Tumour site		0.000		0.000
Whole stomach	1 (Ref)		1 (Ref)	
Upper stomach	0.499 (0.399–0.624)	0.000	1.097 (0.828–1.453)	0.519
Middle stomach	0.324 (0.253–0.415)	0.000	0.919 (0.691–1.223)	0.562
Lower stomach	0.316 (0.263–0.379)	0.000	0.749 (0.5814–0.966)	0.026
> 2/3 stomach	0.512 (0.420–0.623)	0.000	0.774 (0.6110–0.979)	0.033
Gross appearance		0.000		0.000
Borrmann types I	1 (Ref)		1 (Ref)	
Borrmann types II	0.553 (0.331–0.924)	0.024	0.562 (0.331–0.954)	0.033
Borrmann types III	0.864 (0.527–1.417)	0.563	0.833 (0.498–1.392)	0.485
Borrmann types IV	1.856 (1.116–3.087)	0.017	0.970 (0.571–1.648)	0.911
Tumour stage		0.000		0.002
T1	1 (Ref)		1 (Ref)	
T2	8.192 (5.560–12.069)	0.000	3.897 (1.716–8.850)	0.001
T3	15.017 (10.151–22.216)	0.000	4.409 (1.894–10.262)	0.001
T4a	21.388 (14.039–32.585)	0.000	5.901 (2.433–14.317)	0.000
T4b	31.140 (20.876–46.452)	0.000	5.720 (2.382–13.734)	0.000
Lymph-node stage		0.000		0.003
N0	1 (Ref)		1 (Ref)	
N1	1.710 (1.443–2.026)	0.000	1.042 (0.839–1.294)	0.710
N2	2.163 (1.847–2.535)	0.000	1.061 (0.831–1.354)	0.636
N3	3.462 (2.976–4.027)	0.000	1.462 (1.122–1.905)	0.005
TNM stage		0.000		0.000
IA	1 (Ref)		1 (Ref)	
IB	5.046 (3.140–8.110)	0.000	1.095 (0.402–2.984)	0.859
IIA	7.889 (4.966–12.531)	0.000	1.397 (0.503–3.881)	0.521
IIB	11.514 (7.265–18.250)	0.000	1.709 (0.594–4.913)	0.320
IIIA	15.752 (9.982–24.857)	0.000	1.830 (0.627–5.337)	0.269
IIIB	18.880 (11.853–30.074)	0.000	1.775 (0.595–5.296)	0.304
IIIC	34.931 (21.597–56.497)	0.000	2.016 (0.641–6.346)	0.231
IV	45.506 (28.699–72.155)	0.000	1.593 (0.455–5.580)	0.467
Surgery		0.000		0.000
Absolutely curative	1 (Ref)		1 (Ref)	
Relatively curative	2.025 (1.763–2.325)	0.000	1.203 (1.030–1.406)	0.020
Palliative	5.815 (5.051–6.693)	0.000	2.422 (1.755–3.341)	0.000
Lymph node dissection		0.000		0.150
D1	1 (Ref)		1 (Ref)	
D2	0.867 (0.711–1.058)	0.161	0.815 (0.652–1.019)	0.072
D3	0.839 (0.639–1.101)	0.206	0.830 (0.615–1.119)	0.221
Palliative resection	3.323 (2.687–4.111)	0.000	0.677 (0.502–0.914)	0.011
Joint organ removal		0.000		0.020
None	1 (Ref)		1 (Ref)	
Pancreas or spleen	2.125 (1.744–2.590)	0.000	1.229 (0.972–1.553)	0.085
Liver or gall	1.722 (1.291–2.296)	0.000	1.093 (0.773–1.546)	0.615
Transverse colon	2.227 (1.879–2.641)	0.000	1.300 (1.061–1.593)	0.011
Other	2.907 (2.206–3.830)	0.000	1.278 (0.947–1.724)	0.109
Gastrectomy		0.000		0.603
Total	1 (Ref)		1 (Ref)	
Subtotal	0.522 (0.457–0.596)	0.000	1.050 (0.874–1.261)	0.603
Hepatic metastasis		0.000		0.037
No	1 (Ref)		1 (Ref)	
Yes	4.548 (3.555–5.818)	0.000	1.769 (1.035–3.023)	0.037
Peritoneum metastasis		0.000		0.004
No	1 (Ref)		1 (Ref)	
Yes	4.190 (3.547–4.948)	0.000	1.525 (0.837–2.780)	0.168
Adjunctive therapy		0.000		0.001
No	1 (Ref)		1 (Ref)	
Yes	0.720 (0.612–0.846)	0.000	0.766 (0.638–0.919)	0.004
Diagnosis period		0.004		0.023
1980s	1 (Ref)		1 (Ref)	
1990s	0.948 (0.817–1.100)	0.479	0.903 (0.767–1.063)	0.220
2000s	0.823 (0.714–0.948)	0.007	0.846 (0.702–1.020)	0.080

*Ref* Reference category

^a^Derived from tests of HR for prognostic factors in univariate model adjusted for treatment group in Cox proportional-hazards model

^b^Cox-regression analysis, controlling for prognostic factors listed in table

### Computer-aided unsupervised clustering: tumor size

**Fig. 1:**
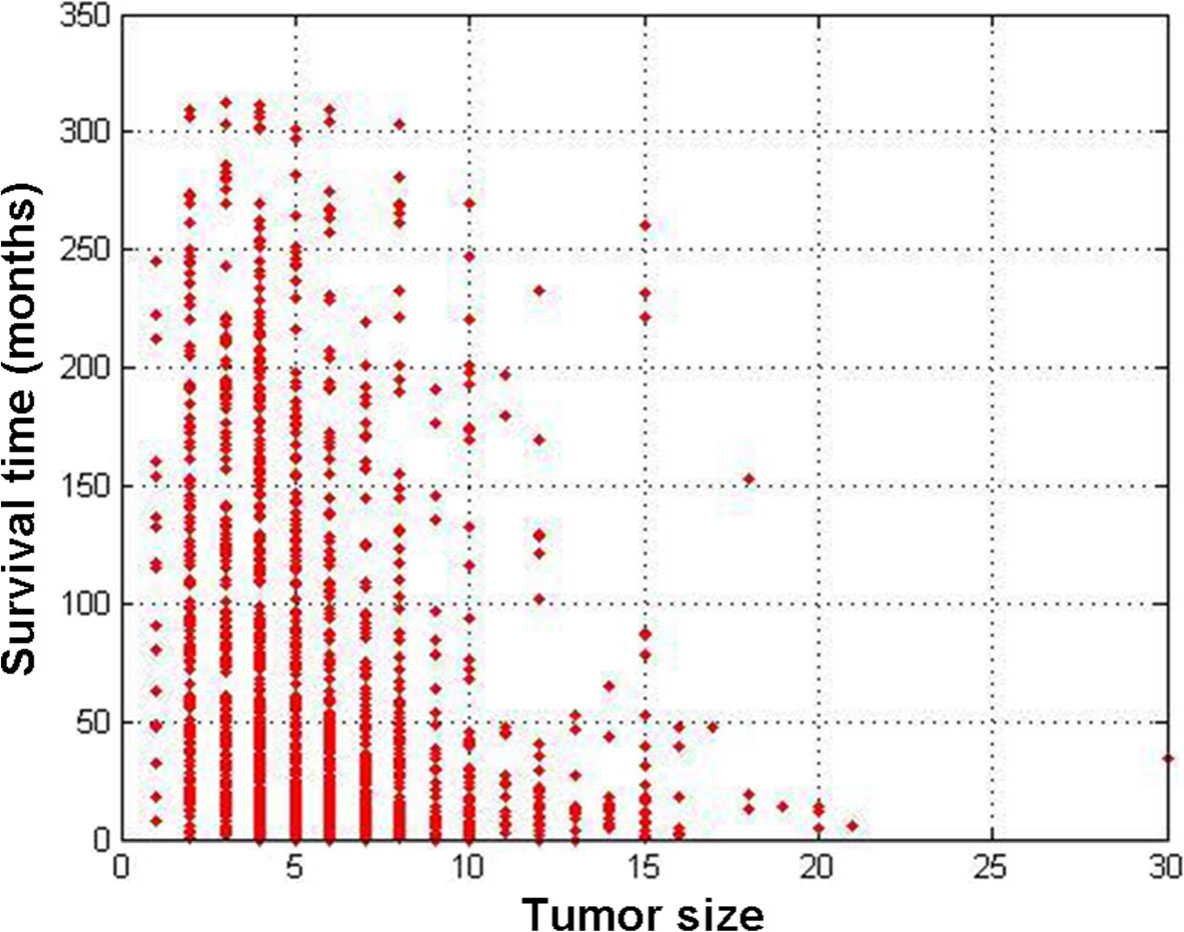
Scatter distribution of tumor size vs. survival time in patients with gastric cancer

**Fig. 2:**
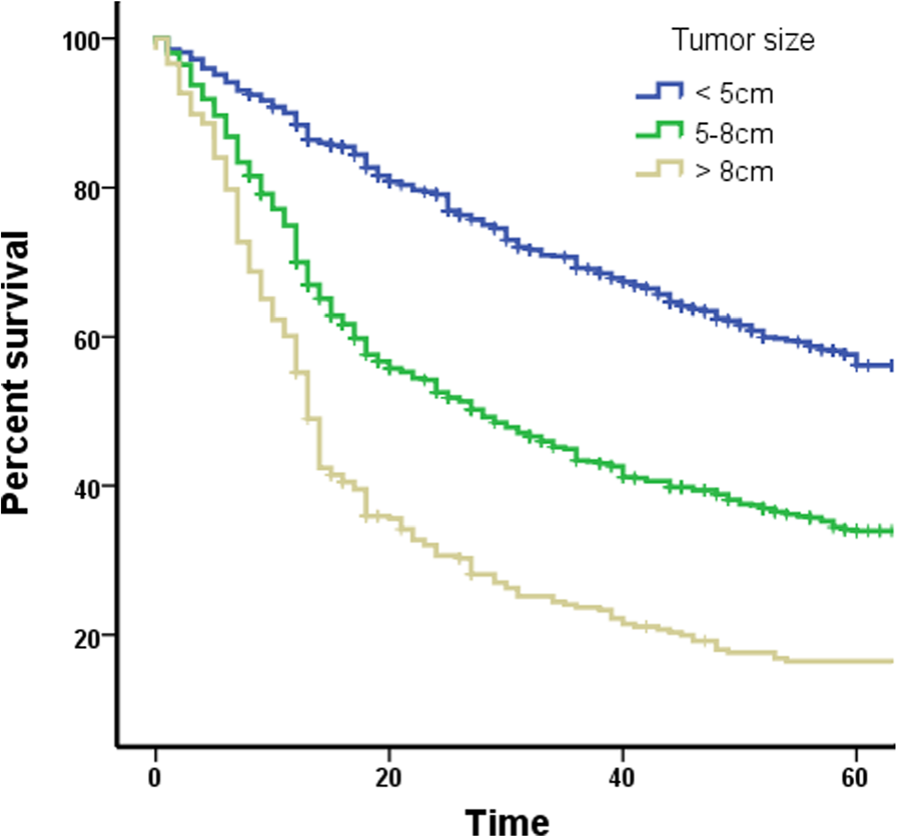
Survival curves according to tumor size in patients with gastric cancer

Patient’s tumor size and survival time were inputted on a dot plot (Fig. 1). After calculations, 5 cm and 9 cm were chosen as the optimal cut-off points, and tumor size was defined as S1 (< 5 cm), S2 (5–8 cm), S3 (≥9 cm), according to when the differences between the groups were maximized (Fig. 2, *p* < 0.001).

### Computer-aided unsupervised clustering: number of lymph node metastases

**Fig. 3:**
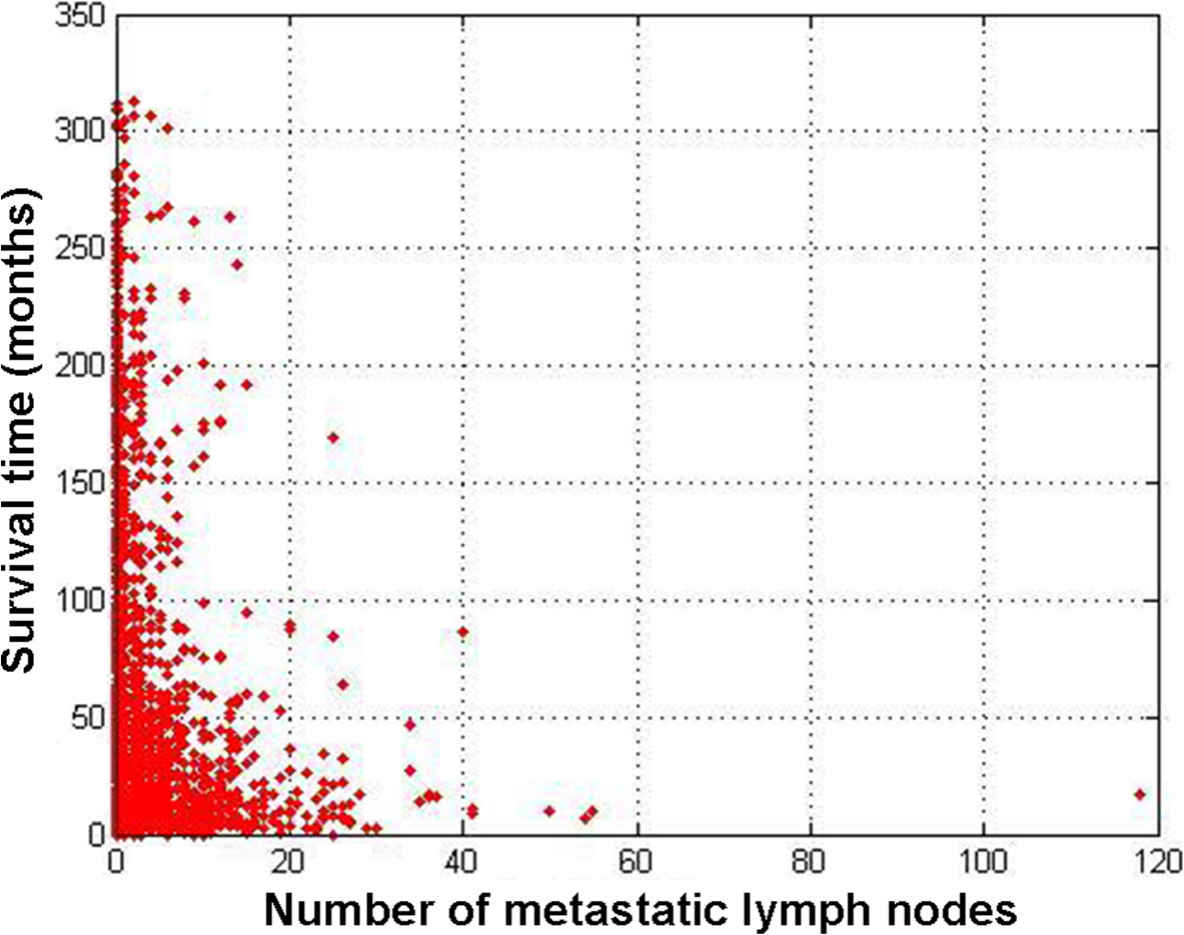
Scatter distribution of the number of lymph node metastases vs. survival time in patients with gastric cancer

**Fig. 4:**
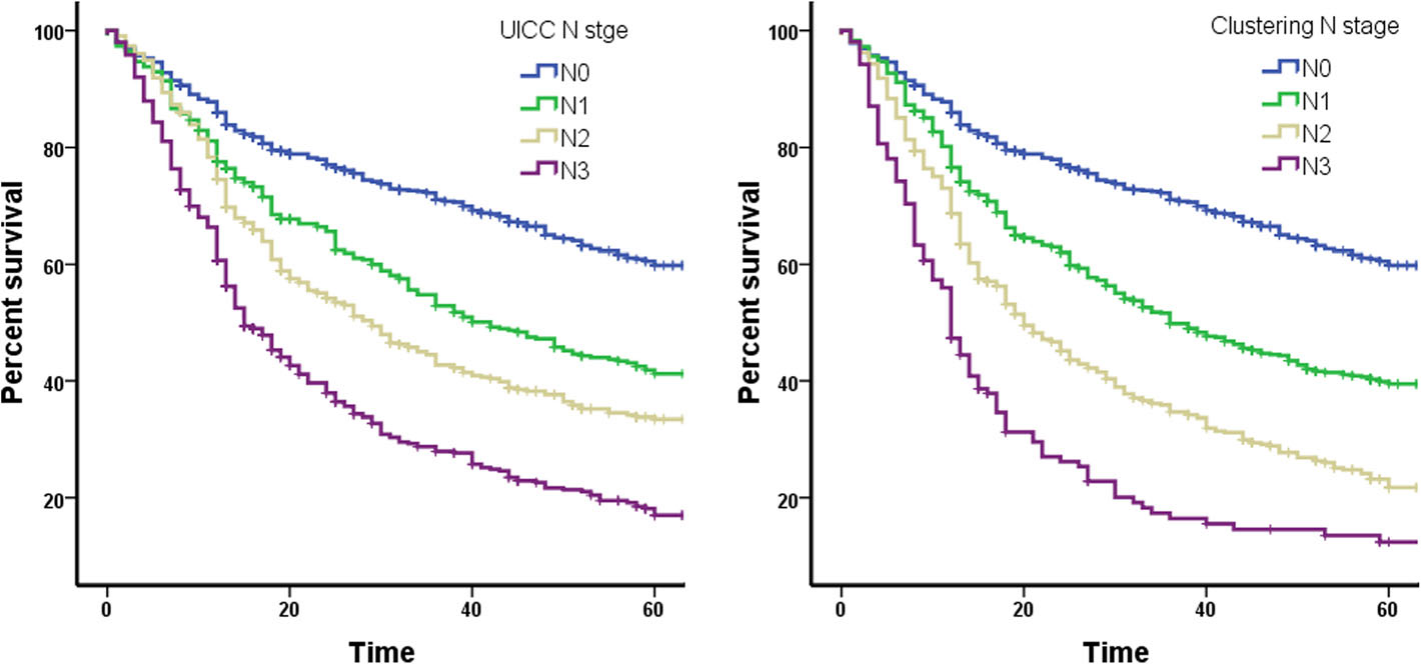
Comparison of survival curves for the clustered N stage and the UICC N stage

Patient number of lymph node metastases and survival time were inputted on a dot plot (Fig. 3). After calculations, 0, 5, and 15 were chosen as the optimal cut-off points and N stages were subdivided as N0 (*n* = 0), N1 (*n* = 1–4), N2 (*n* = 5–14), and N3 (*n* ≥ 15), according to when the differences between the groups were maximized (Fig. 4, *p* < 0.001).

### Computer-aided unsupervised clustering: TNM stage

Based on patients’ prognoses data, the computer-aided unsupervised clustering method was applied to re-cluster patients with different TNM stages. Clustering results and the number of patients in each group after clustering are shown in Table 3, which is also thought as the new TNM staging criteria. In the original 7th edition of the UICC gastric cancer TNM stages, there was an orderly arrangement of the different T, N, and M stages, which was disrupted after computer-aided unsupervised clustering.

**Table 3:** Comparison of the 7th UICC and the clustering TNM stage

UICC						Clustering						
	No.	TNM					No.	TNM				
IA	272	(100)^1^				IA	340	(100)^1^	(110)^3^	(120)^6^		
IB	394	(200)^2^	(110)^3^			IB	363	(200)^2^				
IIA	391	(300)^4^	(210)^5^	(120)^6^		IIA	558	(210)^5^	(220)^9^	(410)^11^	(500)^14^	
IIB	371	(400)^7^	(310)^8^	(220)^9^	(130)^10^	IIB	301	(300)^4^	(310)^8^	(320)^12^		
IIIA	399	(410)^11^	(320)^12^	(230)^13^		IIIA	453	(400)^7^	(130)^10^	(230)^13^	(330)^17^	(221)^27^
IIIB	237	(500)^14^	(510)^15^	(420)^16^	(330)^17^	IIIB	82	(420)^16^	(530)^19^	(211)^26^	(411)^34^	
IIIC	116	(520)^18^	(530)^19^	(430)^20^		IIIC	199	(520)^18^	(430)^20^	(301)^29^	(311)^30^	(321)^31^
								(331)^32^	(421)^35^	(431)^36^	(501)^37^	(511)^38^
IV	234	(101)^21^	(111)^22^	(121)^23^	(131)^24^	IV	118	(510)^15^	(201)^25^	(521)^39^	(531)^40^	(401)^33^
		(201)^25^	(211)^26^	(221)^27^	(231)^28^							
		(301)^29^	(311)^30^	(321)^31^	(331)^32^							
		(401)^33^	(411)^34^	(421)^35^	(431)^36^							
		(501)^37^	(511)^38^	(521)^39^	(531)^40^							

### Effect of TNM stage on prognosis predictions after unsupervised clustering

**Fig. 5:**
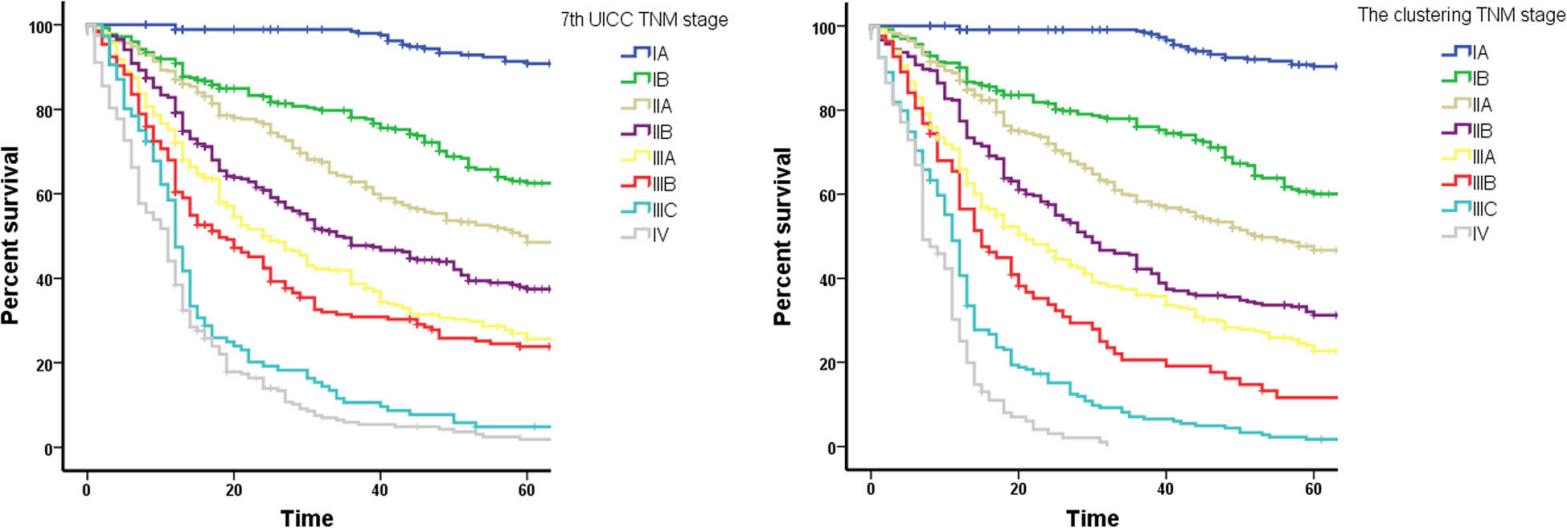
Comparison of survival curves of the clustered TNM stages and the UICC TNM stages

The significance of the differences between the various stages is shown in Table 4. When comparing each row, there was a significant difference between the classes in the clustered stages, making it superior to the UICC staging criteria. Survival rate curves for the 2 different staging methods are shown in Fig. 5. Compared with the UICC stages, which is the “7^th^ UICC TNM stage”, the use of the computerized clustering method, which is the “clustering TNM stage”, resulted in a significant decrease in the differences between the groups for each stage, as well as for the different T and N stages (data not shown).

**Table 4:** Comparison of *P* values between each stage of UICC and the clustering TNM stage

	IA vs. IB	IB vs. IIA	IIA vs. IIB	IIB vs. IIIA	IIIA vs. IIIB	IIIB vs. IIIC	IIIC vs. IV	Average
UICC	2.66e-14	1.79e-04	2.07e-04	1.20e-03	8.61e-02	5.46e-06	2.99e-02	0.0168
Clustering	0	1.00e-04	1.31e-06	2.60e-04	1.73e-02	1.16e-04	161e-04	0.0030

**Fig. 6:**
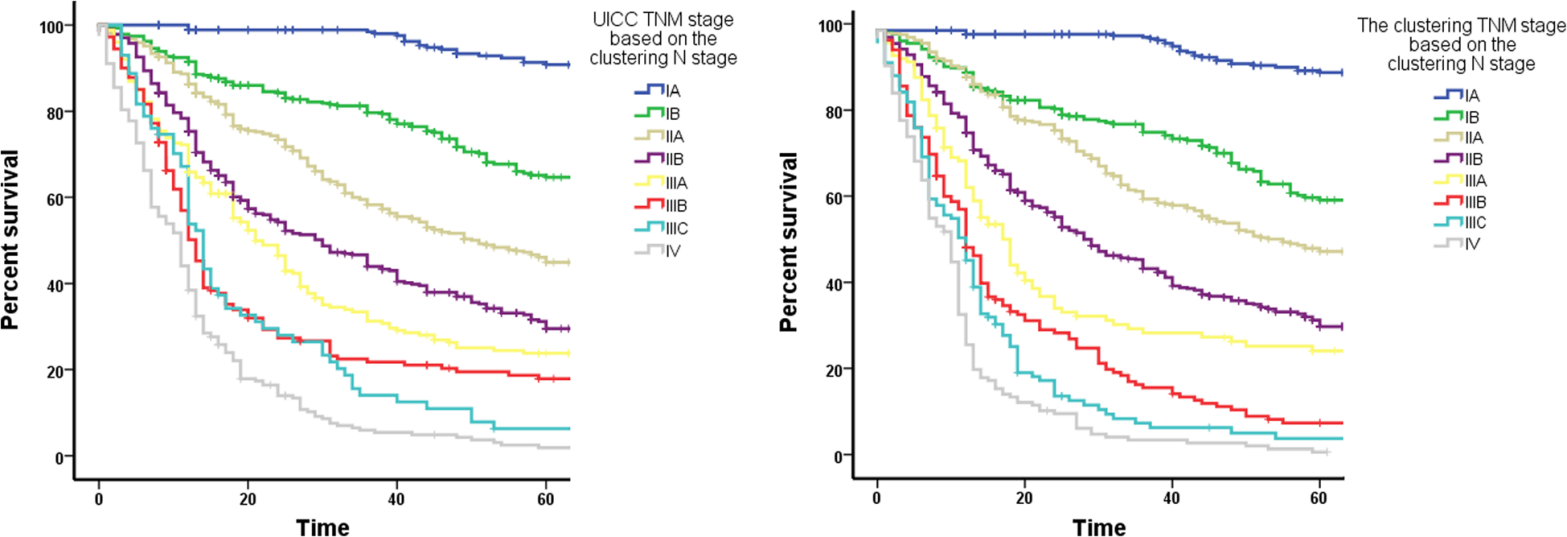
Comparison of survival curves of the clustered TNM stages based on the clustered N stage and the UICC TNM stages based on the clustered N stage

Because we performed clustering analysis on N stage in this study, the N stage of many patients was changed. We also introduced the clustering N stages of N0 (*n* = 0), N1 (*n* = 1–4), N2 (*n* = 5–14), and N3 (*n* ≥ 15) into the UICC TNM stage, which is “the UICC TNM stage based on the clustering N stage” in Fig. 6, and re-performed the unsupervised clustering for TNM stage, which is “the clustering TNM stage based on the clustering N stage”. Survival rate curves for the 2 different staging methods are shown in Fig. 6.

## Discussion

In the past, when performing confirmation or exploratory TNM staging improvements, differences in survival were always compared between different stages by observer-determined divisions. Such methods could result in selection bias, thereby introducing problems in obtaining accurate staging for a particular patient population. However, in computer-aided unsupervised clustering, which is based on patient survival data, patients are clustered inversely. This ensures the accuracy of the patient population for each stage, produces the least amount of heterogeneity between patients, and maximizes survival differences between each stage. Regarding the degree of difference between the classes, although the UICC and Japanese staging criteria have significantly different *p*-values that are superior to the cluster staging method, as a whole, there is a greater degree of difference between classes in the cluster staging method. Neither the UICC nor Japanese criteria consider significant differences between groups within the classes. Rather, they take the groups with greater differences and divide them into a separate class. However, by analyzing the degree of difference between groups within classes, the cluster staging method divides the group with the lowest degree of difference into a separate class, thus creating a lesser degree of difference within classes, which is more in line with actual gastric cancer data.

After clustering the TNM stages, we found that there were more pre-IIIA stage patients compared with the UICC staging system, and there was a particularly significant increase in the number of patients with IA stage disease. This shows that in the past, judgments of a good prognosis may have been limited and pessimistic. Therefore, in some patients, prognosis might need to be revisited to formulate a more accurate and rational comprehensive treatment program. After clustering, the T1N1M0 and T1N2M0 patient classes were added to stage IA, which indicates that the invasion depth of gastric cancer might have a greater effect on patient prognosis compared with the extent of lymph node metastases. Furthermore, the adverse effects caused by lymph node metastases in these patients might be more easily controlled through comprehensive treatment.

By contrast, after clustering, there were significantly fewer patients with stage IV gastric cancer. This indicated that, for many patients, the prognosis might be more optimistic than previously considered. However, many of these patients were classified as having stage IIIC disease, which has a 5-year survival rate of < 10%.

Tumor size is directly related to invasion depth and is an independent prognosticator for gastric cancer. Although the existing gastric cancer staging systems do not take tumor size into consideration, we performed cluster analysis on tumor size based on survival data. The results revealed that in our database, 4 cm and 9 cm represented good tumor size threshold values. The adverse effects of a greater tumor size are caused by a greater invasion depth, more extensive lymph node metastases, and a greater possibility of distant metastases, although they might also be related to the need for a greater extent of gastric resection and the possibility of resection of adjacent organs. Furthermore, in the present study, the median tumor size was ~ 5 cm, indicating that significant improvements are needed regarding gastric cancer screening and early diagnosis. The majority of patients with gastric cancer are elderly and from rural areas, and the lack of timely and standardized treatments, in addition to poor compliance, remain significantly severe issues for interventions [15].

In 2010, the UICC and Japanese TNM staging systems came to an agreement on the divisions for N stage according to the number of lymph node metastases. In the present study, a cluster analysis of the number of lymph node metastases (0, 5, and 15 nodes), based on survival data, improved the distinction of patients’ prognoses compared with the existing classification systems. However, to maintain consistency with the existing UICC stages, when performing multivariate analysis, we did not use the cluster analysis division criteria for N stage and TNM stage analyses.

For cluster analysis according to age, 55 years was found to be optimum age for distinguishing patients’ prognoses. Further subgroup analysis including sex, revealed that in female patients, prognoses could not be divided based on significant differences in critical age values, whereas in male patients, the critical age was 53 years. Therefore, in male patients aged > 53 years, there was a significant difference in diagnosis compared with male patients aged < 53 years. The specific mechanism behind this prognostic difference remains unknown, but this phenomenon might provide clues regarding the pathogenesis of gastric cancer between the sexes.

Because the present study was retrospective, the reliability of the data would be inferior to that obtained in prospective clinical trials; therefore, appropriate TNM classification guidelines for gastric cancer, especially in the Chinese population, need to be studied further. Meanwhile, China is an expansive region where people from different areas have different economic circumstances and lifestyle habits, which has certain effects on the development, progression, and outcome of cancer. In the present study, most of our patients are from northeastern China, which is representative of the characteristics of gastric cancer patients in northeastern China to a certain extent, however, not patients in all of China. In future studies we will increase collaboration with hospitals in other regions to investigate staging methods more appropriate to Chinese patients and behavioral characteristics with respect to gastric cancer biology. Nevertheless, these findings provide a reference for the future improvement of gastric cancer TNM staging, accurate determination of gastric cancer prognoses, and improved implementation of more comprehensive treatments.

## Conclusions

Compared with the existing TNM staging classification for gastric cancer, there was a greater difference between stage classes when using the computer-aided unsupervised clustering method. In addition, in the cluster staging method, groups with a lesser degree of difference were divided into separate classes, thereby creating a staging system that is more in line with actual gastric cancer data. In summary, in Chinese patients with gastric cancer, the cluster staging method was preferable over the UICC or Japanese TNM classification for determining prognosis regarding the degree of difference within classes or among groups within the classes.

## Abbreviations

TNM: Tumor-node-metastasis
UICC: The Union for International Cancer Control

## References

[1] Chae S, Lee A, Lee JH (2011). The effectiveness of the new (7th) UICC N classification in the prognosis evaluation of gastric cancer patients: a comparative study between the 5th/6th and 7th UICC N classification. Gastric Cancer.

[2] Ramadori G, Triebel J (2008). Nodal dissection for gastric cancer. N Engl J Med.

[3] Santiago JM, Sasako M, Osorio J (2011). TNM-7th edition 2009 (UICC/AJCC) and Japanese classification 2010 in gastric Cancer. Towards simplicity and standardisation in the management of gastric cancer. Cir Esp.

[4] Lu J (2013). Consideration of tumor size improves the accuracy of TNM predictions in patients with gastric cancer after curative gastrectomy. Surg Oncol.

[5] Luo Y (2016). Clinicopathologic characteristics and prognosis of Borrmann type IV gastric cancer: a meta-analysis. World J Surg Oncol.

[6] Paoletti X, Oba K, Burzykowski T, Michiels S, Ohashi Y, Pignon JP, Rougier P, Sakamoto J, Sargent D, Sasako M, van Cutsem E, Buyse M, GASTRIC (Global Advanced/Adjuvant Stomach Tumor Research International Collaboration) Group (2010). Benefit of adjuvant chemotherapy for resectable gastric cancer: a meta-analysis. JAMA.

[7] Fujita T (2009). Gastric cancer. Lancet.

[8] Brower V (2015). Modified gastric cancer chemotherapy: more effective, less toxic. Lancet Oncol..

[9] Cristescu R, Lee J, Nebozhyn M, Kim KM, Ting JC, Wong SS, Liu J, Yue YG, Wang J, Yu K, Ye XS, Do IG, Liu S, Gong L, Fu J, Jin JG, Choi MG, Sohn TS, Lee JH, Bae JM, Kim ST, Park SH, Sohn I, Jung SH, Tan P, Chen R, Hardwick J, Kang WK, Ayers M, Hongyue D, Reinhard C, Loboda A, Kim S, Aggarwal A (2015). Molecular analysis of gastric cancer identifies subtypes associated with distinct clinical outcomes. Nat Med.

[10] Shah MA, Ajani JA (2010). Gastric cancer--an enigmatic and heterogeneous disease. Jama.

[11] Markar SR, Wiggins T, Ni M, Steyerberg EW, Van Lanschot JJ, Sasako M, Hanna GB (2015). Assessment of the quality of surgery within randomised controlled trials for the treatment of gastro-oesophageal cancer: a systematic review. Lancet Oncol..

[12] Nishida T, Doi T (2014). Improving prognosis after surgery for gastric cancer. Lancet Oncol..

[13] Shen L, Shan YS, Hu HM, Price TJ, Sirohi B, Yeh KH, Yang YH, Sano T, Yang HK, Zhang X, Park SR, Fujii M, Kang YK, Chen LT (2013). Management of gastric cancer in Asia: resource-stratified guidelines. Lancet Oncol.

[14] Zhang H (2011). Survival trends in gastric cancer patients of Northeast China. World J Gastroenterol.

[15] Herrero R, Parsonnet J, Greenberg ER (2014). Prevention of gastric cancer. Jama.

